# Winter gifts for river ecosystems: A massive supply of earthworms in early winter

**DOI:** 10.1002/ece3.9620

**Published:** 2022-12-08

**Authors:** Ryo Futamura, Chiharu Furusawa, Hisanori Okamiya

**Affiliations:** ^1^ Graduate School of Environmental Sciences Hokkaido University Takaoka, Tomakomai Japan; ^2^ Graduate School of Environmental Science Hokkaido University Sapporo Japan; ^3^ Field Science Center for Northern Biosphere Hokkaido University Takaoka, Tomakomai Japan; ^4^ Museum of Natural and Environmental History Shizuoka Japan

**Keywords:** aquatic invertebrate, resource subsidy, salmonid, terrestrial earthworm, terrestrial–aquatic linkage, winter ecology

## Abstract

Terrestrial resource pulses can significantly affect the community dynamics of freshwater ecosystems. Previously, its effect on the river community is considered to be stronger in summer, whereas weaker in winter when terrestrial invertebrates are less abundant. The movement of the terrestrial earthworms is triggered in winter, so they may be supplied to winter rivers as terrestrial resource pulse, but little is known about it. Here, we report that the massive numbers of the terrestrial earthworms were supplied intensively to an upstream of the small river in early winter. In particular, we found large numbers of megascolecid earthworms were supplied in an upstream of the small river in Northern Japan. Furthermore, we observed that supplied earthworms were consumed by salmonid fish species (masu salmon, white spotted char and rainbow trout) and aquatic invertebrates (gammarid amphipod, planarian flatworm, and stonefly larvae). These findings suggest that the terrestrial earthworms may play a key role in ecosystem functioning in winter when severe and other resources are scarce.

## Graphical abstract

We report that massive numbers of terrestrial earthworms were supplied intensively to an upstream of the small river in early winter. Furthermore, supplied earthworms were consumed by salmonid fish species (masu salmon, white spotted char, and rainbow trout) and aquatic invertebrates (gammarid amphipod, planarian flatworm, and stonefly larvae). These findings suggest that terrestrial earthworms may play a key role in ecosystem functioning in winter when severe and other resources are scarce.

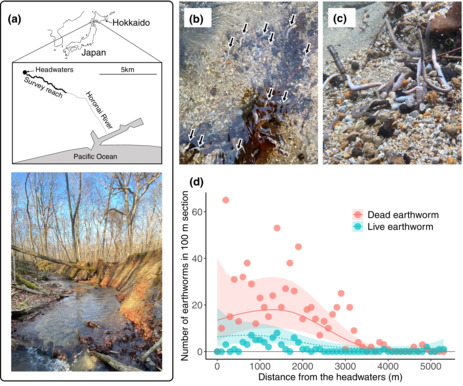

## 1 INTRODUCTION

Terrestrial resource pulses, such as large inputs of arthropods into streams (Nakano et al., 1999), can have significant effects on the community dynamics of freshwater ecosystems through energy flows (Richardson & Sato, 2015). The effect of a terrestrial resource pulse on a freshwater ecosystem varies temporally and spatially (Collins et al., 2016; Leroux & Loreau, 2012). Typically, its effect on river communities is considered to be stronger in summer, when terrestrial invertebrates are more abundant, and weaker in winter, when they are less abundant (Nakano & Murakami, 2001).

Terrestrial earthworms are abundant in soils throughout the world and are high‐energy food for various organisms (Baubet et al., 2003; Macdonald, 1983). The movement of many earthworms is triggered in early winter when the temperature drops steeply (Friend, 1921; Kobayashi et al., 2015), and they may enter rivers as a terrestrial resource pulse. However, to the best of our knowledge, only one study has reported the mass movement of terrestrial earthworms into streams in early winter (Kobayashi et al., 2015), and their utilization in streams has not been examined. Here, we report that terrestrial earthworms were supplied in a large number to the upper reaches of a small river in northern Japan in early winter and were consumed by aquatic organisms.

**FIGURE 1:**
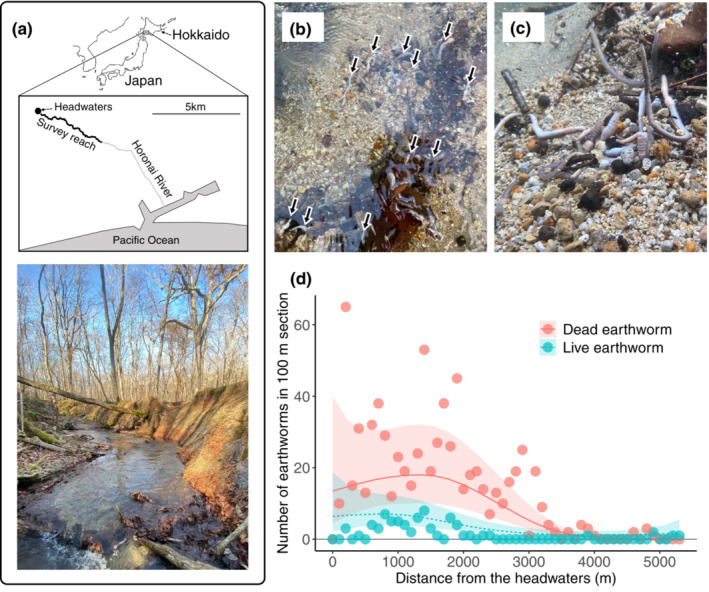
(a) Map and representative photograph of the survey reach in the Horonai River. The survey reach (the uppermost 5320 m of the river) is shown in bold. (b) Terrestrial earthworms lying on the riverbed (black arrows). (c) Earthworms accumulating in a slow‐flowing pool. (d) Distribution of dead (red) and live (blue) worms along the survey reach: Each point indicates the total number of worms found in each 100‐m section. The regression curve was fitted using a generalized additive model. Shading area shows the 95% confidence interval.

In the upper reaches of the Horonai River, a small spring‐fed stream in northern Japan (42°40′N, 141°35′E; Figure 1a), we observed an enormous number of terrestrial earthworms (megascolecid earthworms) in late November 2021 (Figure 1b,c) during a fish tracking survey. Most seemed to be dead, some were fragmented, but some were still alive underwater. To count them, we walked through the uppermost 5320‐m river reach (the survey reach) on 5 December 2021 (Figure 1a). Further downstream was difficult to count them because of its riverscape (see Futamura et al., 2022 for detail). We walked upstream and counted the dead and live worms in each 100‐m section. Since some dead worms were fragmented, we counted only carcass with a visible clitellum. Because the worms' spatial distribution seemed to be non‐linear, we modeled the number of found dead and live earthworms separately by using a generalized additive model, with river section (i.e., distance from the headwaters) as a predictor, assuming negative binomial distribution and log‐link function. Statistical analysis was conducted in the “mgcv” package of R v. 4.1.2 software (R Core Team, 2021).

In total, we found 740 dead and 71 live earthworms in the 5320‐m survey reach. Their mean number of worms found per 100‐m section was 15 ± 16.34 (mean ± SD). The largest number was 68, found at 200–300 m from the headwaters. The live earthworms seemed to have been supplied to the river within 1 day, because megascolecid earthworms die within several hours after entering the water (Chuang & Chen, 2008). Both dead and live worms were found intensively between the headwaters and the middle reach (Figure 1d) (GAM, *p* < .001; Appendix S1: Table S1). This result indicates that dead worms were not transported further downstream but were supplied intensively near the headwaters. We also note that the movement of these earthworms was not triggered by precipitation because there was no rain within a week.

**FIGURE 2:**
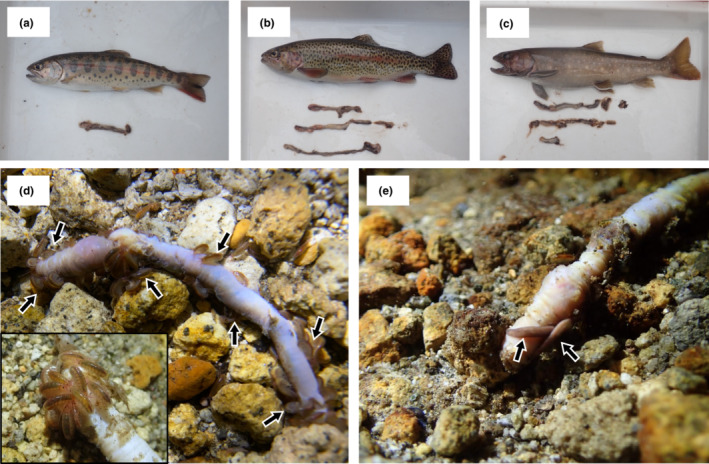
Aquatic organisms that had consumed terrestrial earthworms. Salmonid fish species: (a) masu salmon (*Oncorhynchus masou*), (b) rainbow trout (*O. mykiss*), (c) white‐spotted char (*Salvelinus leucomaenis*). Benthic invertebrates (black arrows), (d) gammarid amphipod (Amphipoda), and (e) planarian flatworm (Tricladida).

We observed aquatic organisms consuming the worms in the upper reaches of the river. During a fish gut contents survey conducted on 11 December 2021, we found three salmonid species that had consumed the worms: masu salmon (*Oncorhynchus masou*; Figure 2a), rainbow trout (*Oncorhynchus mykiss*; Figure 2b), and white‐spotted char (*Salvelinus leucomaenis*; Figure 2c). The proportion of the individual consuming worm was 57% (4/7), 75% (6/8), and 100% (2/2) for masu salmon, rainbow trout and white‐spotted char, respectively. Although a previous study showed that rainbow trout consumes earthworms in spring to autumn (Kawaguchi et al., 2007), it remained unknown whether fish consume them in winter. Here, we provide rare evidence that fish do, in fact, consume earthworms in early winter. Additionally, we observed aquatic invertebrates such as gammarid amphipoda (Amphipoda; Figure 2d), planarian flatworms (Tricladida; Figure 2e), and stonefly larvae (Plecoptera) consuming them on 7 December 2021. For aquatic invertebrates, we could not quantify how often they consume worms.

Previous studies have shown that the terrestrial resource pulses affect the community dynamics of the freshwater ecosystems all over the world (i.e., from sub‐arctic to trophic areas) (Nakano & Murakami, 2001; Recalde et al., 2016; Richardson & Sato, 2015; Romero et al., 2016; Wallace et al., 1997). However, to best to our knowledge, no studies have shown that their effects were strong in severe seasons such as winter in sub‐arctic areas. Furthermore, previous studies have focused on terrestrial arthropods, and other terrestrial invertebrates such as earthworms have received little attention. Here, we show that a large number of earthworms were supplied intensively to the upper reaches of a small river during early winter, and several aquatic organisms were consuming them. This result suggests that terrestrial earthworms play an important role in ecosystem functioning in a freshwater ecosystem in winter, when other food items are scarce.

One of our key findings is that aquatic invertebrates were consuming the worms. Many studies have documented that freshwater fish do so (Inoue et al., 2013; Itakura et al., 2021; Kawaguchi et al., 2007; Mason & MacDonald, 1982), but, to the best of our knowledge, no studies have shown utilization of earthworms by aquatic invertebrates. Although we could identify aquatic invertebrate only to order level due to its difficulty, we observed a broad range of aquatic invertebrate taxa consuming the worms as allochthonous prey. Although terrestrial invertebrate inputs indirectly affect the aquatic invertebrate community by altering the intensity of fish predation in the food web (Nakano et al., 1999), their direct effects remain unclear. This massive supply of earthworms in winter provides a unique opportunity to examine this topic. Further studies should identify the taxonomy of aquatic invertebrates that consume worms in lower levels (e.g., genus level) and quantify its utilization of terrestrial resources by aquatic invertebrates to reveal the effect of terrestrial resource pulses on freshwater ecosystems.

Why and how did the earthworms enter the river in winter? It is possible that they were accidentally supplied to the river from the riverbank where the soil was exposed. Although the behavioral mechanism is unknown, their movement is triggered in early winter, when the temperature drops and the soil moisture declines (Friend, 1921). This could cause some to inadvertently fall from the riverbank into the river, in particular from steep sites where the soil is exposed. Our additional data support this idea, as we found denuded slopes more frequently in the upper reaches of the stream (Appendix S1: Figure S1), corresponding with the distribution of earthworm supply to the river. Because denuded slopes are often concreted by revetments worldwide (Grill et al., 2019), river development may have decreased the supply of worms to the river in early winter.

Large numbers of earthworms appear to be supplied continually and broadly in early winter. In the Horonai River, they seemed to be supplied for at least a few weeks, because we observed them from 18 November until 11 December and could not observed it after 11 December 2022 (personal observation). They appear to be supplied also to other rivers in northern Japan in early winter. A previous study reported that enormous amounts of terrestrial earthworms were supplied to the small Doran River (44°48′N, 142°06′E) in early winter (Kobayashi et al., 2015). We also observed earthworms entering the Yufutsu River, an adjacent tributary of the Horonai River (42°42′N, 141°33′E), on 11 December 2021 (personal observation). Thus, the terrestrial earthworm supply could be a common phenomenon in early winter and may play an important role in northern temperate freshwater ecosystems.

In summary, we found that many terrestrial earthworms were supplied to the upper reaches of a river in early winter and aquatic organisms were consuming them. Winter is severe for aquatic organisms when prey availability is low and metabolic costs are high (Huusko et al., 2007; Sutton et al., 2021). Thus, storing energy before winter is key to survival during winter (Hurst, 2007). Seasonal access to high‐energy prey in early winter could provide some aquatic organisms with substantial energy benefits to survive the winter, shaping survivorship and having spillover effects on populations, communities, and ecosystem functioning. In particular, intense earthworm subsidies to the upper stream may increase the heterogeneity of consumer abundance along a stream gradient and shape the food web structure. Our observation emphasizes the need for further studies on the effects of terrestrial resource pulses on freshwater ecosystems, especially in winter, to deepen our understanding of terrestrial–aquatic linkages.

## AUTHOR CONTRIBUTIONS

**Ryo Futamura:** Conceptualization (equal); data curation (equal); formal analysis (equal); funding acquisition (lead); investigation (equal); methodology (equal); project administration (equal); writing – original draft (equal). **Chiharu Furusawa:** Conceptualization (equal); data curation (equal); investigation (equal); writing – original draft (equal). **Hisanori Okamiya:** Conceptualization (equal); data curation (equal); formal analysis (equal); investigation (equal); methodology (equal); writing – original draft (equal).

## CONFLICT OF INTEREST

We have no conflict of interest.

## Supporting information


Appendix S1

## Data Availability

The data is available at figshare (https://doi.org/10.6084/m9.figshare.21647300.v1).
