# Supplementary material for: Winter gifts for river ecosystems: A massive supply of earthworms in early winter
Source: Ecol Evol. 2022 Dec 8;12(12):e9620. doi: 10.1002/ece3.9620 (PMC9732096; doi:10.1002/ece3.9620)

# Additional Supporting Information 1

**Table S1** Results of a generalized additive model

|  | E.df | Ref.df | Chi.sq | *P*-value |
| --- | --- | --- | --- | --- |
| (1) Distribution of dead worms |  |  |  |  |
| s (Section) | 3.364 | 4.192 | 102.4 | <0.001 |
| (2) Distribution of live worms |  |  |  |  |
| s (Section) | 4.731 | 5.752 | 46.74 | <0.001 |

Abbreviations: E.df, Estimated degrees of freedom; Ref.df, Reference degrees of freedom; Chi.sq, Chi-squared value

**Figure S1** Spatial distribution of denuded slopes along the survey reach. Each point indicates a denuded slope in a 100-m river section (1 = denuded slope, 0 = no denuded slope). The regression curve was fitted using logistic regression. The probability of occurrence of a denuded slope was significantly higher upstream than downstream (Survey reach, χ^2^ = 11.51, *P* < 0.001). Gray shading shows the 95% confidence interval.


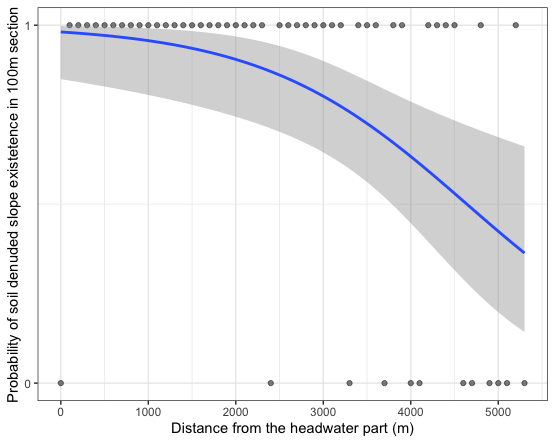

Supplement: Supplementary file 1 — Appendix S1 [file ECE3-12-e9620-s001.docx]
